# Supplementary material for: HIVprotI: an integrated web based platform for prediction and design of HIV proteins inhibitors
Source: J Cheminform. 2018 Mar 9;10:12. doi: 10.1186/s13321-018-0266-y (PMC5845081; doi:10.1186/s13321-018-0266-y)
Supplement: Supplementary file 2 — Additional file 2. Source code of HIVProtI web server. [file 13321_2018_266_MOESM2_ESM.zip › HIVprotI_Source-code/hivprotI/weka-3-6-6/doc/weka/datagenerators/classifiers/classification/RandomRBF.html]

RandomRBF


---


|  |  |  |
| --- | --- | --- |
| **Overview** \| **Package** \| **Class** \| **Tree** \| **Deprecated** \| **Index** \| **Help** \| **Weka's home** | |  |
| **PREV CLASS**   **NEXT CLASS** | **FRAMES**    **NO FRAMES**     **All Classes** |  |
| SUMMARY: NESTED \| FIELD \| CONSTR \| METHOD | DETAIL: FIELD \| CONSTR \| METHOD |  |


---


## weka.datagenerators.classifiers.classification Class RandomRBF

```
java.lang.Object
  weka.datagenerators.DataGenerator
      weka.datagenerators.ClassificationGenerator
          weka.datagenerators.classifiers.classification.RandomRBF
```

**All Implemented Interfaces:**: java.io.Serializable, OptionHandler, Randomizable, RevisionHandler

---

``` public class RandomRBF extends ClassificationGenerator ```

RandomRBF data is generated by first creating a random set of centers for each class. Each center is randomly assigned a weight, a central point per attribute, and a standard deviation. To generate new instances, a center is chosen at random taking the weights of each center into consideration. Attribute values are randomly generated and offset from the center, where the overall vector has been scaled so that its length equals a value sampled randomly from the Gaussian distribution of the center. The particular center chosen determines the class of the instance.
